# Screening performance of COPD-PS scale, COPD-SQ scale, peak expiratory flow, and their combinations for chronic obstructive pulmonary disease in the primary healthcare in Haicang District, Xiamen City

**DOI:** 10.3389/fmed.2024.1357077

**Published:** 2024-04-09

**Authors:** Xueting Shen, Hua Yang, Chengdian Lan, Fen Tang, Qinfei Lin, Yingjie Chen, Jinxiang Wu, Xionghua Chen, Zhigang Pan

**Affiliations:** ^1^Department of General Medicine, Zhongshan Hospital, Fudan University, Shanghai, China; ^2^Department of General Medicine, Xiamen Branch, Zhongshan Hospital, Fudan University, Xiamen, China; ^3^Health Bureau of Haicang, Xiamen, China; ^4^Department of General Medicine, Shitang Community Health Service Center, Xiamen, China; ^5^Department of General Medicine, Songyu Community Health Service Center, Xiamen, China; ^6^Department of General Medicine, Xinyang Community Health Service Center, Xiamen, China; ^7^Department of General Medicine, Dongfu Community Health Service Center, Xiamen, China

**Keywords:** chronic obstructive pulmonary disease (COPD), screening test, COPD-SQ questionnaire, COPD-PS questionnaire, peak expiratory flow (PEF), primary healthcare (PHC), diagnosis

## Abstract

**Objectives:**

This study aimed to evaluate the screening performance of COPD-PS questionnaire, COPD-SQ questionnaire, peak expiratory flow (PEF), COPD-PS questionnaire combined with PEF, and COPD-SQ questionnaire combined with PEF for chronic obstructive pulmonary disease (COPD).

**Methods:**

This was a cross-sectional study. We distributed self-designed surveys and COPD screening scales (COPD-PS questionnaire and COPD-SQ questionnaire) to residents who underwent physical examination in five community health centers in Haicang District, Xiamen City, from February 2023 to May 2023, and measured their lung function and PEF with a portable device. We used logistic regression to obtain the coefficients of COPD-PS questionnaire, COPD-SQ questionnaire, and PEF, and plotted the receiver operating characteristic curves of each tool for diagnosing COPD and moderate-to-severe COPD. We evaluated and compared the optimal cut-off points and scores of sensitivity, specificity, Youden index, and area under the curve (AUC) values, and assessed the screening efficiency of different methods.

**Results:**

Of the 3,537 residents who completed the COPD-SQ questionnaire, COPD-PS questionnaire, and spirometry, 840 were diagnosed with COPD. We obtained the coefficients of COPD-PS questionnaire combined with peak expiratory flow (PEF), and COPD-SQ questionnaire combined with PEF, by logistic regression as −0.479-0.358 × PEF +0.321 × COPD-PS score and − 1.286-0.315 × PEF +0.125 × COPD-SQ score, respectively. The sensitivity of diagnosing COPD by COPD-SQ questionnaire, COPD-PS questionnaire, PEF, COPD-PS questionnaire combined with PEF, and COPD-SQ questionnaire combined with PEF were 0.439, 0.586, 0.519, 0.586, 0.612 respectively, and the specificity were 0.725, 0.621, 0.688, 0.689, 0.663 respectively, with ROC values of 0.606 (95%CI: 0.586–0.626), 0.640 (0.619–0.661), 0.641 (0.619–0.663), 0.678 (0.657–0.699), 0.685 (0.664–0.706) respectively. The sensitivity of diagnosing GOLD II and above by COPD-SQ questionnaire, COPD-PS questionnaire, PEF, COPD-PS questionnaire combined with PEF, and COPD-SQ questionnaire combined with PEF were 0.489, 0.620, 0.665, 0.630, 0.781 respectively, and the specificity were 0.714, 0.603, 0.700, 0.811, 0.629 respectively, with ROC values of 0.631 (95%CI: 0.606–0.655), 0.653 (0.626–0.679), 0.753 (0.730–0.777), 0.784 (0.762–0.806), 0.766 (0.744–0.789) respectively.

**Conclusion:**

Our study found that the accuracy of COPD screening by COPD-SQ questionnaire and COPD-PS questionnaire can be improved by combining the results of PEF. The screening performance of COPD-SQ questionnaire combined with PEF is relatively better. In future research, further studies are needed to optimize the performance of screening tools and understand whether their use will affect clinical outcomes.

## 1 Introduction

Chronic obstructive pulmonary disease (COPD) is a common, preventable, and treatable chronic airway disease that causes irreversible airway obstruction and progressive lung function decline (1). Patients with COPD often remain undiagnosed for about 3.6 ± 4 years after the onset of symptoms, leading to clinical deterioration such as worsening lung function, increased symptoms, or acute exacerbations (2). Early diagnosis of patients with irreversible obstruction by spirometry, irrespective of their symptoms, is essential to prevent further lung damage and improve their quality of life. Spirometry is the “gold standard” for diagnosing COPD and evaluating its severity, progression, prognosis, and treatment response (3). However, spirometry is not widely available or accessible in many settings, and it requires time, effort, and proper patient cooperation to obtain accurate results (4). Therefore, a simple, easy-to-use, low-cost and time-efficient screening method is needed to identify patients at high risk of COPD who would benefit from spirometry testing (5). Family doctors can provide follow-up care and treatment to control symptoms and slow down disease progression for patients with early COPD screened out. Patients with moderate or severe COPD, however, need more frequent medical monitoring and intervention because they have higher rates of lung function decline, acute exacerbation, and mortality than those in the early stage. Therefore, screening for this type of patient is more important.

Martinez et al. developed a three-level screening strategy based on the “COPD assessment in primary care to identify undiagnosed respiratory disease and exacerbation risk questionnaire” (CAPTURE), which can reduce the workload while ensuring the screening efficiency, and has reference value^[6]^. However, the CAPTURE questionnaire performed poorly in primary care settings in China (6, 7). However, the CAPTURE questionnaire performed poorly in primary care settings in China (8) The “Expert Consensus on COPD Screening in County-level Areas of China (2020)” recommended that different regions and units can choose COPD-PS or COPD-SQ for COPD screening according to their own needs (8). However, the criteria for selecting the suitable questionnaire based on “own needs” are unclear, and primary care physicians face a challenge in making this decision. Moreover, the current screening tools and methods are mainly applied for research purposes, and their effectiveness in real-world settings is not well reported.

Therefore, we performed questionnaire screening and PFT among residents who underwent physical examination in Haicang District, Xiamen City. Our aim was to compare the screening performance of COPD-PS questionnaire, COPD-SQ questionnaire, peak expiratory flow (PEF), COPD-PS questionnaire plus PEF, and COPD-SQ questionnaire plus PEF for detecting COPD by PFT.

## 2 Materials and methods

### 2.1 Participants

This study is a cross-sectional study. The study population comprised residents who underwent physical examinations at five community health service centers (Shitang, Dongfu, Songyu, Qiaonan, and Xinyang) located in Haicang District, Xiamen City, between February 2023 and May 2023. The inclusion criteria for this study required participants to be aged ≥40 years and permanent residents of Haicang District, Xiamen City.

The exclusion criteria were as follows: (1) Individuals who have suffered from myocardial infarction, stroke, or shock in the past three months. (2) Individuals with severe heart failure, severe arrhythmia, or unstable angina within the past four weeks. (3) Individuals who have experienced massive hemoptysis within the past four weeks. (4) Individuals who require medication for epilepsy. (5) Patients with uncontrolled primary hypertension (systolic blood pressure > 200 mmHg and/or diastolic blood pressure > 100 mmHg; 1 mmHg = 0.133 kPa). (6) Patients with aortic aneurysm. (7) Patients with severe hyperthyroidism. (8) Individuals with respiratory system diseases other than chronic obstructive pulmonary disease, such as bronchiectasis, bronchial asthma, lung cancer, and respiratory infectious diseases. (9) Individuals who have recently undergone eye, ear, or cranial surgery. (10) Individuals with pneumothorax or giant bulla who are not ready for surgical treatment. (11) Pregnant women. (12) Individuals with psychiatric disorders or cognitive impairments.

### 2.2 Research tools

#### 2.2.1 Portable spirometer

This study used a portable spirometer (X1, Xeek, Co., Ltd., Xiamen, China) to measure lung function. This device is a small and low-cost instrument that can measure lung function easily and portably. It is based on the physical principle of differential pressure transducer, and measures the flow and volume of gas exhaled or inhaled by patients through the respiratory pipeline. It can measure conventional ventilation function parameters, such as forced vital capacity (FVC), forced expiratory volume in 1 s (FEV_1_), peak expiratory flow (PEF), etc., and can perform bronchodilation test. A previous study compared the consistency of this portable spirometer with a standard spirometer, and found no significant bias and good agreement for all parameters. It is suitable for clinical applications such as primary lung function screening, COPD diagnosis and treatment, lung function follow-up of patients in remote areas, etc. (9).

#### 2.2.2 The “COPD population screener”

The COPD-PS questionnaire, developed by the American Clinical Practice Group in 2008, is the most common screening tool in primary health care settings in China (10). It consists of five items that assess objective factors (age and smoking status) and subjective symptoms (dyspnea, activity change, and cough/sputum). Each item is scored from 0 to 2 points, and the total score ranges from 0 to 10 points. A higher score indicates a higher likelihood of having COPD (11). Current guidelines and literature recommend pulmonary function screening for residents with a COPD-PS score of 5 or more to confirm the diagnosis of COPD (11, 12).

#### 2.2.3 The “COPD self-screening questionnaire”

The COPD-SQ questionnaire is a screening tool for COPD adapted and validated by Chinese scholars for the Chinese population. It has seven questions that evaluate subjective symptoms (cough and dyspnea on weekdays) and objective factors (age, smoking status, body mass index, family history, and biomass fuel exposure) (13). The developers of the COPD-SQ questionnaire emphasized that biomass smoke is a major risk factor for female COPD, and it is important to include it in the screening questionnaire (13). Each item has different scores depending on the options, and the total score ranges from 0 to 28 points. A higher score indicates a higher probability of having COPD. PFT is advised for patients with a total score of 16 or more to confirm the diagnosis of COPD. The COPD-SQ questionnaire differs from the COPD-PS questionnaire by adding the assessment of biomass fuel exposure, which is a significant risk factor for COPD in Chinese women.

#### 2.2.4 Peak expiratory flow

PEF is the maximum flow at the mouth achieved during an expiration, delivered with maximum force starting from the level of maximum lung inflation (14). It is a common measure of pulmonary ventilation function that correlates well with FEV1 measured by spirometry and reflects airway patency (14). PEF also has a good correlation with the St. George’s Respiratory Questionnaire score and indicates the quality of life of patients (15). PEF only requires a short maximum expiration time and less operation skills, and has high repeatability and user compliance. It can be used as an effective tool for identifying COPD patients, monitoring and predicting COPD acute exacerbations (16).

### 2.3 Research methods

#### 2.3.1 Questionnaire completion

The primary health care physicians in Haicang District, Xiamen City, were trained uniformly by Xiamen Hospital affiliated to Zhongshan Hospital of Fudan University.

With the help of the healthcare physicians, the residents filled out a self-designed general information questionnaire and the COPD-PS and COPD-SQ questionnaires on the spot. The healthcare physicians checked and collected the questionnaires. For those who had difficulty in completing the questionnaires, the healthcare physicians explained and filled them out for them.

#### 2.3.2 Pulmonary function testing

We used a portable spirometer (model: X1, China Xeek company) to measure the lung function parameters (FEV_1_, FVC, PEF, etc.) of the community residents, with the assistance of primary community physicians who had received professional training. The bronchodilation test was performed 15 min after inhaling salbutamol 200ug via a metered-dose inhaler. We followed the quality control standards of the American Thoracic Society (ATS) and performed up to eight tests before and after bronchodilation. We obtained at least two ATS-acceptable pulmonary function curves, and the variation of FEV_1_ and FVC between the two best and largest tests was <0.2 L, or < 0.1 L when FVC < 1 L (17). We assigned the quality control level (A-F) according to the quality control standards, and only pulmonary function tests of level A, B, and C were included in the analysis. All subjects were asked to sit, pinch their nose, and use a disposable mouthpiece.

#### 2.3.3 COPD diagnosis and grading criteria

According to the 2023 GOLD guidelines (1), COPD is diagnosed as FEV_1_/FVC < 0.70 after bronchodilation test. The bronchodilation test was positive if FEV_1_ increased by more than 15% or more than 0.2 L after inhaling salbutamol 200ug for 15 min, compared with baseline. Among patients with FEV_1_/FVC < 0.7, the severity of COPD is graded according to the percentage of FEV_1_ to the predicted FEV_1_ (FEV1%pred), that is, FEV_1_%pred≥80% is GOLD I (mild), 80%>FEV_1_%pred≥50% is GOLD II (moderate), 50%>FEV_1_%pred≥30% is GOLD III (severe), and FEV_1_%pred<30% is GOLD IV (very severe).

#### 2.3.4 The smoking index

The smoking index was obtained by multiplying the daily cigarette consumption/exposure and the smoking duration in years. Smoking index/secondhand smoke index ≤200 was mild smoke, 201–400 was moderate smoking smoke, and >400 was heavy smoking smoke (18).

### 2.4 Quality control

We trained the investigators and pulmonary function testers uniformly before conducting the formal research survey. They had to pass five qualified tests in the pre-survey. All operations (questionnaire, pulmonary function testing, review and revision, etc.) were signed by operators. We randomly selected 10% of the pulmonary function test results for review by two pulmonary function experts who agreed on the diagnosis. We uploaded all questionnaire data and pulmonary function results to the Xeek intelligent management platform cloud backup, which prevented any deletion or modification.

### 2.5 Statistics analysis

Descriptive analysis was employed to evaluate demographic characteristics, spirometric parameters, and questionnaire scores. Continuous variables were reported as mean ± standard deviation. The optimal cut-off point for the screening questionnaire was determined by selecting the value with the highest Youden’s index. A multiple logistic regression was run with COPD as a dependent variable, while the independent variables included the combined COPD-PS scale with PEF and COPD-SQ scale with PEF. The receiver operating characteristic (ROC) curve was plotted for each screening method. Sensitivity, specificity, Youden’s index, and the area under the receiver operating characteristic curve (AUROC) were calculated for the optimal cut-off value and previously recommended values. Statistical significance was set at *p* < 0.05. All analyses were performed by R v4.3.1 (R Core Team, Vienna, Austria).

## 3 Results

### 3.1 Demographic and related information

**Figure 1:**
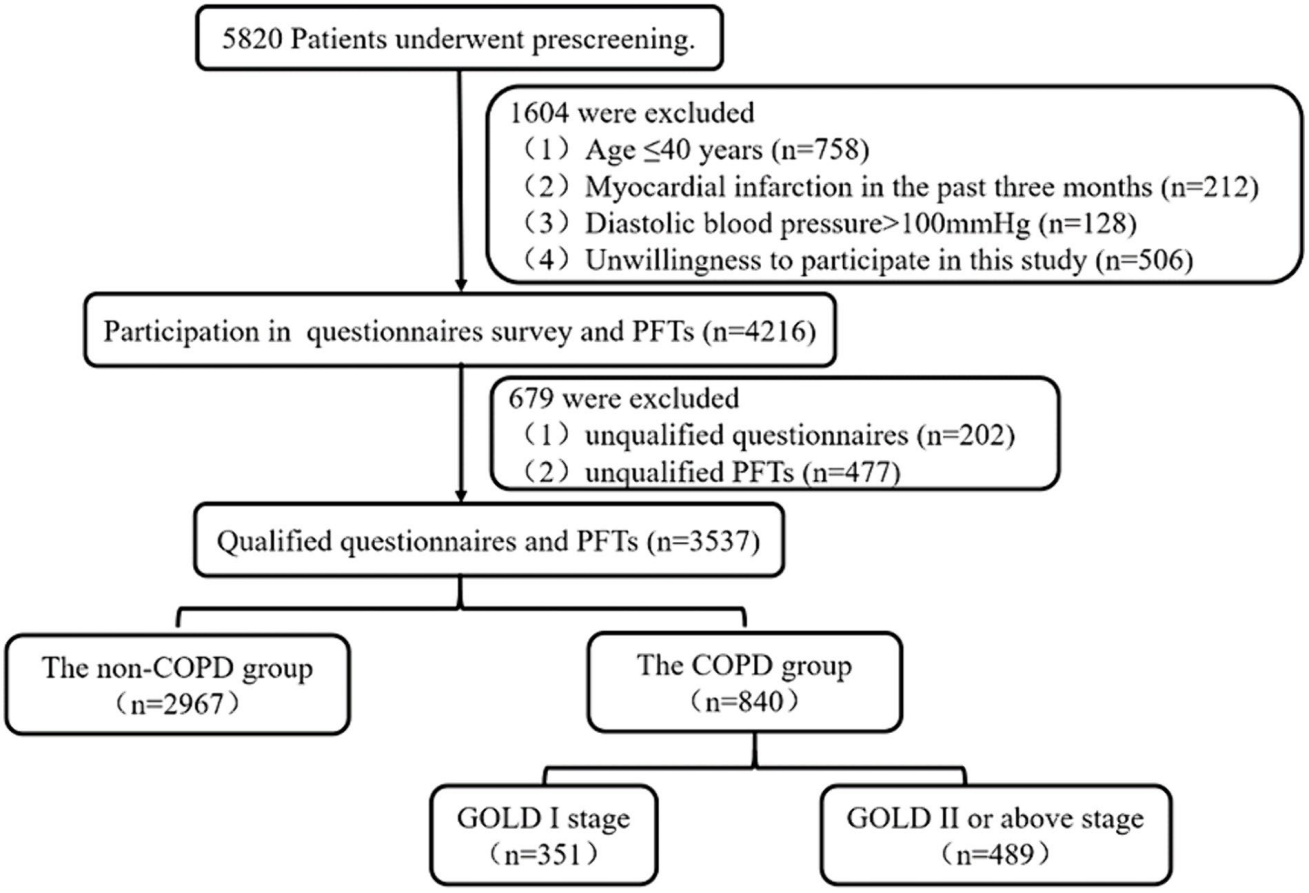
The flowchart of study. COPD, chronic obstructive pulmonary disease; GOLD, Global Initiative for Chronic Obstructive Lung Disease; PFTs, pulmonary function tests.

This study involved 4,216 residents, of whom 3,537 completed both a valid questionnaire survey and spirometry, yielding an effective rate of 83.9%. Figure 1 shows the flow chart of the study. A total of 840 subjects (455 males) were diagnosed with chronic obstructive pulmonary disease. Table 1 presents the demographic characteristics, spirometry results, and screening questionnaire results.

**Table 1:** Demographic and related information between the COPD group and the non-COPD group.

	The total (*n* = 3,537)	The COPD group (*n* = 840)	The non-COPD group (*n* = 2,697)	*p*	GOLD I (*n* = 351)	GOLD II, III and IV(n = 489)	*p*
Gender	−	−	−	0.000	−	−	0.000
Male	1,537	455	1,082	−	174	281	−
Female	2000	385	1,615	−	177	208	−
Age	66.29 ± 7.89	68.28 ± 6.86	65.67 ± 8.09	0.000	66.27 ± 7.92	66.29 ± 7.90	0.000
40–49	186	12	174	−	6	9	−
50–59	364	63	301	−	32	31	−
60–69	1887	427	1,460	−	185	242	−
70–79	1,030	315	715	−	125	190	−
≥80	60	23	47	−	3	17	−
BMI (kg/m^2^)	24.65 ± 3.18	23.97 ± 3.04	24.85 ± 3.19	0.000	24.67 ± 3.18	23.94 ± 3.09	0.000
<18.5	62	17	45	−	5	12	−
18.5–24.9	1,468	432	1,036	−	186	246	−
25–29.9	1,507	310	1,197	−	128	182	−
>30	500	87	419	−	32	49	−
PFT	−	−	−	−	−	−	−
PEF, L/s	4.47 ± 1.54	3.90 ± 1.48	4.64 ± 1.51	−	4.49 ± 1.54	3.33 ± 1.25	−
FVC, L	2.69 ± 0.80	2.83 ± 0.81	2.64 ± 0.79	−	2.68 ± 0.80	2.46 ± 0.65	−
FEV_1_, L	2.02 ± 0.60	1.80 ± 0.57	2.08 ± 0.59	−	2.02 ± 0.60	1.50 ± 0.43	−
FEV_1_, %pred	84.56 ± 18.22	74.54 ± 19.20	87.68 ± 16.21	−	84.95 ± 18.12	61.96 ± 13.91	−
FEV_1_/FVC, %	75.65 ± 9.67	63.18 ± 6.87	79.54 ± 6.65	−	76.02 ± 9.33	61.01 ± 0.08	−
GOLD stage	−	−	−	−	−	−	−
GOLD I	−	351	−	−	−	−	−
GOLD II	−	394	−	−	−	−	−
GOLD III	−	77	−	−	−	−	−
GOLD IV	−	18	−	−	−	−	−
Smoking index	−	−	−	0.000	−	−	0.000
Nonsmoker	2,913	623	2,290	−	283	340	−
Mild	97	31	66	−	9	22	−
Moderate	129	42	87	−	16	26	−
Severe	398	144	254	−	43	101	−
Secondhand smoking index	−	−	−	0.001	−	−	0.000
Nonsmoker	3,045	696	2,394	−	298	398	−
Mild	146	38	108	−	18	20	−
Moderate	97	22	75	−	6	16	−
Severe	249	84	165	−	29	55	−
Biomass Combustion	−	−	−	0.119	−	−	0.190
Yes	714	188	526	−	274	110	−
No	2,823	652	2,171	−	77	379	−
Family history of COPD	−	−	−	0.005	−	−	0.000
Yes	168	55	113	−	11	45	−
No	3,369	785	2,584	−	340	444	−
Previously diagnosis of COPD	−	−	−	0.000	−	−	0.000
Yes	36	21	15	−	2	19	−
No	3,501	819	2,682	−	349	470	−
Previously diagnosis of tuberculosis	−	−	−	0.001	−	−	0.087
Yes	36	17	19	−	8	9	−
No	3,501	823	2,687	−	343	480	−
Previously SARS-CoV-2 infected	−	−	−	0.000			0.001
Yes	2,642	571	2071	-	236	335	−
No	895	269	626	-	115	154	−
Complications	−	−	−	−			−
Hypertension	1,534	329	1,205	0.005	126	203	0.399
Diabetes	606	113	493	0.001	47	66	0.025
Coronary heart disease	67	25	42	0.357	9	16	0.026
Gout	59	17	42	0.008	7	10	0.609
Scale Scores	−	−	−	−	−	−	
COPD-PS	2.47 ± 1.26	2.87 ± 3.54	2.44 ± 1.27	0.000	2.45 ± 1.26	3.06 ± 1.46	
COPD-SQ	11.07 ± 3.92	12.61 ± 4.95	10.71 ± 3.96	0.000	11.03 ± 3.92	12.97 ± 4.00	

COPD, chronic obstructive pulmonary disease; BMI, body mass index; PFT, pulmonary function test; PEF, Peak expiratory flow; FVC, forced vital capacity; FEV_1_, forced expiratory volume in 1 s; % pred, % predicted; GOLD, Global Initiative for Chronic Obstructive Lung Disease; COPD-PS, the “COPD Population Screener”; COPD-SQ, the “COPD Self-Screening Questionnaire” (COPD-SQ).

### 3.2 Multiple logistic regression analysis

We performed a multifactor logistic regression to examine the association between COPD presence and the COPD-PS score, the COPD-SQ score, and the PEF results. The results showed that lower PEF results, higher COPD-PS scores, and higher COPD-SQ scores were significantly associated with increased COPD risk. The regression equations were:

$$\left[\mathrm{Logit}\left(\mathrm{COPDPS}+PEF\right)\right]=-0.479-\left(0.358\times PEF\right)+\left(0.321\times \mathrm{COPDPS}\right)$$

$$\left[\mathrm{Logit}\left(\mathrm{COPDSQ}+PEF\right)\right]=-1.286-\left(0.315\times PEF\right)+\left(0.125\times \mathrm{COPDSQ}\right)$$

Tables 2, 3 show the logistic regression results of the COPD-PS score and the COPD-SQ score, respectively, in combination with the PEF.

**Table 2:** Results of logistic regression analysis of COPD-PS score and PEF.

Variables	β	SE	Wald	OR	*p*
COPDPS	0.321	0.032	100.558	1.379 (1.295, 1.468)	0.000
PEF	−0.358	0.030	142.207	0.699 (0.659, 0.741)	0.000
Constant	−0.479	0.150	10.137	0.620	0.001

COPD-PS, the “COPD Population Screener”; PEF, Peak expiratory flow.

**Table 3:** Results of logistic regression analysis of COPD-SQ score and PEF.

Variables	β	SE	Wald	OR	*p*
COPDSQ	0.125	0.011	120.403	1.134 (1.109, 1.159)	0.000
PEF	−0.315	0.030	109.907	0.730 (0.688, 0.774)	0.000
Constant	−1.286	0.195	43.376	0.276	0.000

COPD-SQ, the “COPD Self-Screening Questionnaire” (COPD-SQ); PEF, Peak expiratory flow.

### 3.3 The performance of different screening methods for COPD

**Figure 2:**
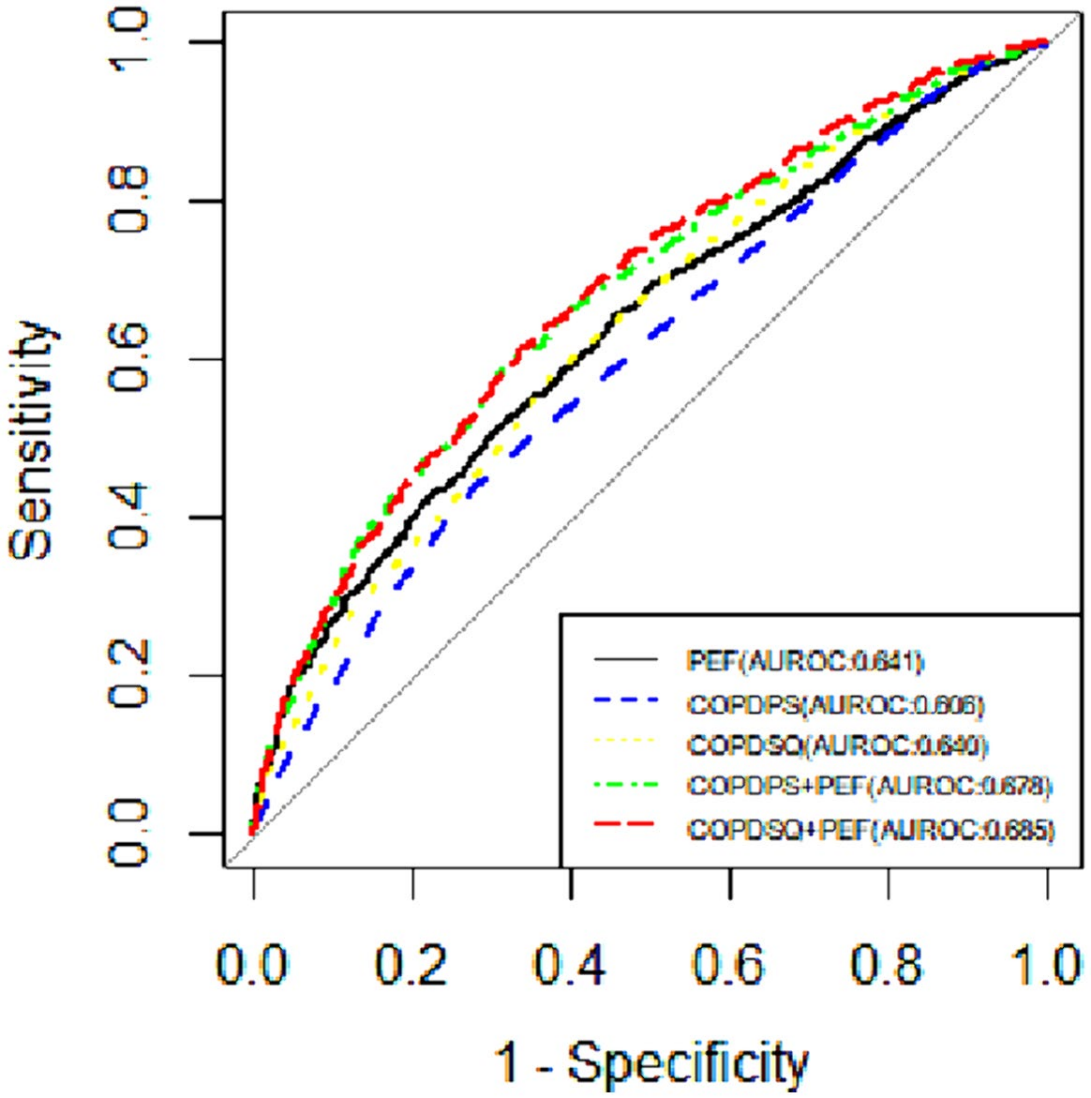
The ROC curves of different screening methods for diagnosing COPD. COPD-PS, the “COPD Population Screener”; COPD-SQ, the “COPD Self-Screening Questionnaire” (COPD-SQ); PEF, Peak expiratory flow.

We calculated the sensitivity, specificity, Youden index and ROC of five screening methods for COPD: COPD-PS scale, COPD-SQ scale, PEF, COPD-PS scale combined with PEF and COPD-SQ scale combined with PEF. We reported the optimal cut-off values for each method. Table 4 shows the performance of different screening methods for COPD with different cut-off values. Figure 2 shows the ROC curves of each screening method.

**Table 4:** The performance of different screening methods for COPD.

	Optimal cut-off	Sensitivity	Specificity	Youden index	PPV	NPV	ROC (95%CI)
COPD-PS	2.5	0.439	0.725	0.164	0.332	0.806	0.606 (0.586–0.626)
COPD-SQ	11.5	0.586	0.621	0.207	0.325	0.828	0.640 (0.619–0.661)
PEF^$^	3.765	0.519	0.688	0.207	0.341	0.821	0.641 (0.619–0.663)
COPD-PS + PEF^*^	−1.058	0.586	0.689	0.275	0.37	0.842	0.678 (0.657–0.699)
COPD-SQ + PEF^#^	−1.079	0.612	0.663	0.275	0.361	0.846	0.685 (0.664–0.706)

$Since PEF is negatively correlated with COPD, the ROC calculation in this study used negative PEF; * COPD-PS + PEF = [Logit(COPD-PS + PEF)] = −0.479 – (0.358 * PEF) + (0.321 * COPD-PS); ^#^COPD-SQ + PEF = [Logit(COPD-SQ + PEF)] = −1.286 – (0.315 * PEF) + (0.125 * COPD-SQ); COPD-PS, the “COPD Population Screener”; COPD-SQ, the “COPD Self-Screening Questionnaire” (COPD-SQ); PEF, Peak expiratory flow; PPV, Positive predictive value; NPV, Negative Predictive Value.

### 3.4 The performance of different screening methods for moderate and above COPD

**Figure 3:**
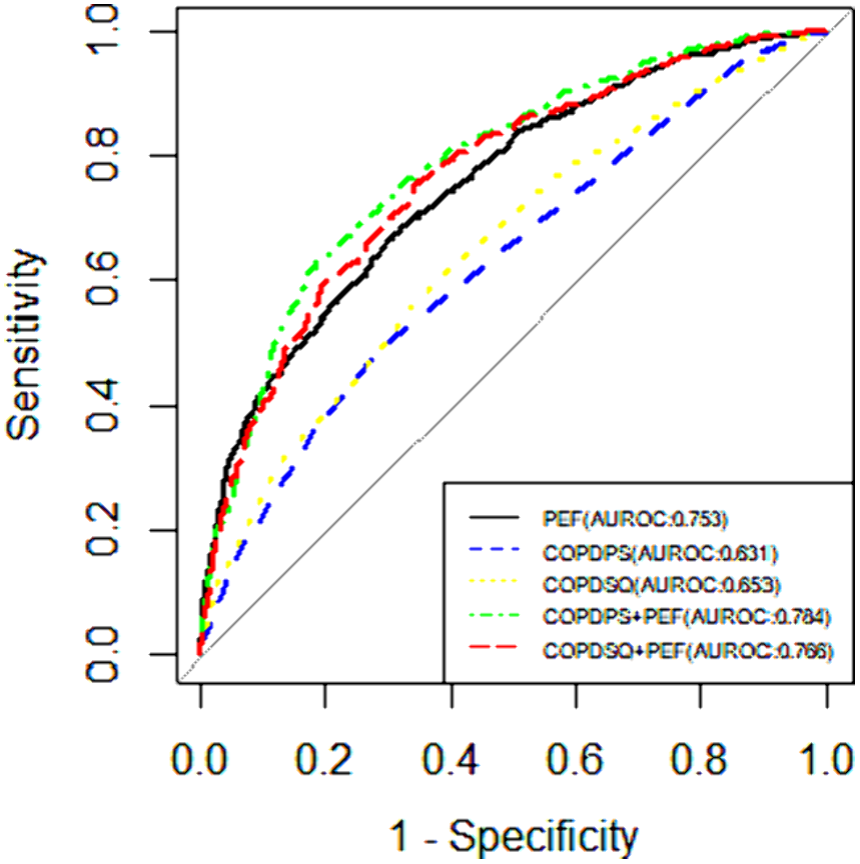
The ROC curves of different screening methods for diagnosing moderate and above COPD. COPD-PS, the “COPD Population Screener”; COPD-SQ, the “COPD Self-Screening Questionnaire” (COPD-SQ); PEF, Peak expiratory flow.

We evaluated the sensitivity, specificity, Youden index and ROC of five screening methods for COPD and reported the optimal cut-off values for each method. Table 5 shows the performance of different screening methods for moderate and above COPD with different cut-off values. Figure 3 shows the ROC curves of each screening method for diagnosing moderate and above COPD.

**Table 5:** The performance of different screening methods for moderate and above COPD.

	Optimal cut-off	Sensitivity	Specificity	Youden index	PPV	NPV	ROC (95%CI)
COPD-PS	2.5	0.489	0.714	0.203	0.215	0.897	0.631 (0.606–0.655)
COPD-SQ	11.5	0.620	0.603	0.223	0.200	0.908	0.653 (0.626–0.679)
PEF	3.725	0.665	0.700	0.365	0.262	0.929	0.753 (0.730–0.777)
COPD-PS + PEF	−0.844	0.630	0.811	0.441	0.349	0.932	0.784 (0.762–0.806)
COPD-SQ + PEF	−1.122	0.781	0.629	0.410	0.252	0.947	0.766 (0.744–0.789)

COPD-PS, the “COPD Population Screener”; COPD-SQ, the “COPD Self-Screening Questionnaire” (COPD-SQ); PEF, Peak expiratory flow.

## 4 Discussion

This study aimed to evaluate the screening effects of COPD-PS questionnaire, COPD-SQ questionnaire, PEF, COPD-PS questionnaire + PEF and COPD-SQ + PEF for chronic obstructive pulmonary disease (COPD). The results showed that all five methods had some screening ability for COPD and moderate or above COPD among residents in Haicang District of Xiamen City, China, and that adding PEF improved the performance of the questionnaires. Among the five screening methods, COPD-SQ questionnaire combined with PEF had relatively better diagnostic ability for COPD. However, when screening patients with moderate or above COPD, the sensitivity of COPD-PS questionnaire + PEF and COPD-SQ questionnaire + PEF was (0.630 vs. 0.781), specificity was (0.811 vs. 0.629), Youden index was (0.441 vs. 0.410), and area under the receiver operating characteristic curve (AUROC) was (0.784 vs. 0.766). The purpose of this study was to find a suitable screening method for COPD, which required a balance between sensitivity and specificity. Sensitivity is the ability of the screening test to accurately identify patients with a specified disease, and specificity is the ability of the screening test to accurately identify patients without the disease. High sensitivity results mean that there are few false negative results and missed cases. When the disease is serious and treatable in the pre-clinical stage, sensitivity is usually increased at the expense of specificity to increase the potential screening value (19). Therefore, although COPD-PS questionnaire + PEF had higher Youden index and AUROC than COPD-SQ questionnaire, we suggest using COPD-SQ questionnaire + PEF to screen patients with COPD and moderate or above COPD, considering the preference of screening test for high sensitivity, until further research can potentially optimize the performance of screening tools.

Pulmonary function test (PFT) is the “gold standard” for diagnosing COPD, but it is time-consuming, labor-intensive, and requires trained professionals. Therefore, most COPD screening uses a two-stage strategy: first, a screening questionnaire to assess the risk factors of patients and divide them into “high-risk” and “low-risk” groups; second, a PFT for the high-risk group to confirm the diagnosis of COPD. This strategy is simple, convenient, and more cost-effective than questionnaire or PFT alone. In China, common COPD screening questionnaires include COPD-PS, COPD-SQ, CAPTURE, and COPD-MH. Among them, COPD-PS is currently the most widely used (12, 20). However, some studies in Beijing and Shanghai found that COPD-SQ had a better screening effect than COPD-PS, which was consistent with this study. However, some studies in Beijing and Shanghai found that COPD-SQ had a better screening effect than COPD-PS (17, 21), which was consistent with this study. Moreover, the Chinese National Chronic Obstructive Pulmonary Disease Screening Program also recommends using COPD-SQ for COPD screening in primary healthcare center (18). The CAPTURE questionnaire was developed by Martinez et al. in the US, and it covers exposure to risk factors, respiratory problems, environmental effects, life and work impacts, fatigue, and respiratory diseases (22). Pan et al. conducted a large-scale multicenter study based on primary health care institutions in China, and found that the Youden index of CAPTURE was lower than that of COPD-SQ (0.220 vs. 0.326), suggesting that COPD-SQ had a better screening performance (23). COPD-MH is a questionnaire developed by Shi Jindong et al. based on primary community hospitals in Minhang District in 2022. The results showed that COPD-MH had a better screening effect than both COPD-SQ and COPD-PS (17). However, since COPD-MH was released recently and only studied in Minhang District in Shanghai, its screening effect needs to be further verified. The two-stage screening method also has some limitations. First, the questionnaire may not capture all the characteristics of COPD patients, especially other risk factors in the early stage of COPD. Second, the questionnaire may involve patients recalling past habits, which are subjective and may produce recall bias. In summary, the two-stage screening method is an effective method for COPD screening, but it needs to be constantly adjusted and optimized in practical application.

PEF is a simple, reliable, and low-cost method of lung function testing that measures the highest flow rate during forced expiration and reflects the degree of airflow limitation. PEF are smaller and more portable than spirometers, making them convenient, feasible, and reproducible for screening and monitoring COPD. Previous studies have shown that PEF can predict and detect COPD hospitalization exacerbations (24–27). COPD screening questionnaires assess the likelihood of COPD by asking patients about their symptoms and signs to assess the likelihood of COPD, but this assessment may be biased by subjective factors. PEF, as an objective indicator of lung function, can directly measure the expiratory flow rate and reflect the degree of airflow limitation. Therefore, combining screening questionnaires with PEF can provide more comprehensive and accurate information and improve the screening performance. In 2016, Martinez et al. applied CAPTURE questionnaire and PEF to screen COPD in 346 subjects in US pulmonary and primary care clinics, and constructed a three-level screening strategy of “Questionnaire-PEF-Spirometry” (28). They found that combining PEF increased the AUROC of CAPTURE questionnaire from 0.795 to 0.906. In 2023, they expanded the study and applied CAPTURE questionnaire and PEF to screen COPD in 4658 subjects in primary care clinics. They also found that combining PEF increased the AUROC of CAPTURE questionnaire indeed (28). However, Yang et al. conducted CAPTURE questionnaire, COPD-SQ questionnaire and PEF in residents aged 35 years and above in Beijing primary health care institutions, and found that using COPD-SQ questionnaire alone had the best screening performance, while combining PEF reduced the screening performance of both questionnaires (7). They attributed this contradiction to the epidemic situation or the poor cooperation between the subjects and the spirometry examiners. This study and Martinez et al.’s study both suggested that PEF can be combined with screening questionnaires to improve the screening ability of primary health care institutions for COPD. In future studies, we hope to further explore the value of PEF in COPD screening, diagnosis, follow-up and prognosis, and provide more valuable information for early, comprehensive, individualized treatment and management of COPD.

This study has important significance for screening COPD in primary health care institutions. Spirometry is often difficult to perform in these settings due to the lack of professional personnel and equipment. Therefore, the screening strategy of COPD-SQ questionnaire combined with PEF can provide a simple, fast, low-cost, and efficient method for primary health care institutions, which can help to improve the diagnosis and treatment of COPD, and enhance the prognosis and quality of life of patients. In the future, training and education for medical staff and patients in primary health care institutions can be strengthened to improve their understanding and mastery of PEF usage methods and significance.

This study has limitations. First, the results of screening tools reflect the clinical characteristics of primary health care cohort in Haicang District of Xiamen City. However, the applicability of COPD screening tools may vary in different regions, different age groups, different severity levels, different risk factors, etc. Second, FEV_1_/FVC ratio decreases with age increase, using a fixed cut-off point FEV_1_/FVC < 0.70 to define COPD may overestimate the risk of COPD in elderly subjects (29, 30). In future studies, we suggest conducting research on more regions and larger samples to verify the results of this study. In addition, further optimization of screening tools’ performance is needed, as well as understanding whether their use will affect clinical outcomes.

## 5 Conclusion

Our study found that the accuracy of COPD screening by COPD-SQ questionnaire and COPD-PS questionnaire can be improved by combining the results of PEF. The screening performance of COPD-SQ questionnaire combined with PEF is relatively better. In future research, further studies are needed to optimize the performance of screening tools and understand whether their use will affect clinical outcomes.

## Data availability statement

The datasets presented in this article are not readily available because all data from this project is required to be handled confidentially. To adhere to the confidentiality guidelines provided by our funding agency, interested parties are welcome to contact us via email at shelly1019@126.com where upon proper recording and with the necessary permissions, we can then share the data.

## Ethics statement

The studies involving humans were approved by the Ethics Committee of Xiamen Branch, Zhongshan hospital, Fudan University. The studies were conducted in accordance with the local legislation and institutional requirements. The participants provided their written informed consent to participate in this study.

## Author contributions

XS: Writing – original draft, Writing – review & editing, Conceptualization, Formal analysis. HY: Writing – review & editing. CL: Data curation, Formal analysis, Writing – original draft. FT: Data curation, Supervision, Writing – original draft. QL: Data curation, Supervision, Writing – original draft. YC: Data curation, Supervision, Writing – original draft. JW: Writing – original draft, Data curation. XC: Data curation, Supervision, Writing – original draft. ZP: Conceptualization, Resources, Writing – review & editing.
